# The detection, treatment, and biology of epithelial ovarian cancer

**DOI:** 10.1186/1757-2215-3-8

**Published:** 2010-03-29

**Authors:** Jennifer AA Gubbels, Nick Claussen, Arvinder K Kapur, Joseph P Connor, Manish S Patankar

**Affiliations:** 1 Department of Biology, Augustana College, 2001 S. Summit Ave, Sioux Falls, SD, 57197, USA; 2 Department of Obstetrics and Gynecology, University of Wisconsin-Madison, 600 Highland Ave, Madison, WI, 53792, USA

## Abstract

Ovarian cancer is particularly insidious in nature. Its ability to go undetected until late stages coupled with its non-descript signs and symptoms make it the seventh leading cause of cancer related deaths in women. Additionally, the lack of sensitive diagnostic tools and resistance to widely accepted chemotherapy regimens make ovarian cancer devastating to patients and families and frustrating to medical practitioners and researchers. Here, we provide an in-depth review of the theories describing the origin of ovarian cancer, molecular factors that influence its growth and development, and standard methods for detection and treatment. Special emphasis is focused on interactions between ovarian tumors and the innate and adaptive immune system and attempts that are currently underway to devise novel immunotherapeutic approaches for the treatment of ovarian tumors.

## Ovarian cancer occurrence

Epithelial ovarian cancer (EOC) is the most deadly of gynecological cancers and is the seventh-leading cause of cancer deaths in women. In 2008, there were 21,650 cases reported which resulted in the deaths of 15,520 women in the United States [1]. Spread of the disease within the peritoneal cavity is associated with non-specific clinical symptoms that are often mistaken for other gastrointestinal or reproductive diseases. Some of the most common symptoms are abdominal discomfort and bloating. Other symptoms include vaginal bleeding, gastrointestinal discomfort, early satiety, and urinary tract symptoms [2]. Another obstacle hindering diagnosis is the fact that the ovaries are deep within the pelvic cavity and difficult to palpate, especially in peri-post menopausal women, the group with the highest incidence of the disease. Because of these reasons, 70% of patients are not diagnosed with the disease until the cancer has metastasized beyond the ovaries and is at stage III or IV [3]. However, studies surveying ovarian cancer patients demonstrate that over 95% of EOC patients had abdominal complaints for many months before their diagnosis [4-6]. There is now a new initiative to quantify the symptoms experienced by ovarian cancer patients prior to diagnosis of the disease. A "Symptoms Index" has been established and studies are underway to determine if it can be used- either independently or in combination- with a molecular marker as a predictor of early stage ovarian cancer [5,6].

There are several different types of ovarian cancers depending upon the cell type of origin. Epithelial cell ovarian cancer (EOC) constitutes 90% of ovarian cancers, while gonadal-stromal (6% occurrence), and germ cell (4% occurrence) tumors make up the rest of the incidence of ovarian cancer patients [7]. As ovarian cancer of epithelial cell origin is the most common type, EOC is discussed throughout this review.

The majority of EOC cases are sporadic in nature and occur in women with no known predisposing factors and thus, in the general population, the overall risk of EOC is low (2-5%). Only a small percentage (5-10%) of EOC patients have a genetic predisposition to the disease. Ninety percent of these patients are carriers of mutated BRCA1 and/or BRCA2 genes, which are also implicated in hereditary breast cancer [8]. These genes normally act as tumor suppressors and regulate cellular proliferation and DNA repair by maintaining chromosome integrity. Mutations in these genes render the proteins unable to perform their intended functions. The lifetime risk of ovarian cancer for patients with BRCA1 mutations is 20% to 60%, and the risk for BRCA2 mutation carriers is 10% to 35% [8]. Ovarian cancers associated with germline mutations of BRCA1 appear to be predominantly of serous type and age of the patient at diagnosis is significantly less as compared to the sporadic ovarian cancers [9,10]. Women who have this mutation may elect to undergo prophylactic bilateral salpingo-oophorectomy (removal of both fallopian tubes and ovaries).

## Origins of EOC

The normal ovarian surface epithelium (OSE) covers the surface of the ovary. OSE is highly responsive to environmental stimuli, including those associated with ovulation [11]. In a normal woman, OSE are a monolayered squamous-to-cuboidal epithelium which functions to shuttle molecules in and out of the peritoneal cavity, as well as participates in the rupture and repair that accompanies every ovulation [12]. These cells are morphologically indistinct and histologically simple; therefore, it is difficult to understand how these cells can transform into tumors [13]. The OSE derive from the embryonic celomic epithelial cells which are a part of the mesoderm. The fallopian tube, uterus, and endocervix are derived from the Mullerian duct which is an invagination of the celomic epithelium. It is hypothesized that OSE cells retain the ability to differentiate into four major histological subtypes, which could explain the distinct histological EOC subtypes. There are four common sub-types of EOC including serous (fallopian tube-like), endometrioid (endometrium-like), mucinous (endocervical-like), and clear cell (mesonephros-like) [12].

The differentiation of OSE cells from cuboidal epithelial cells to a mesenchymal phenotype that is characteristic of Mullerian duct-derived tissues is termed epithelial- mesenchymal transition (EMT). The occurrence of EMT is postulated to aid cells in movement during embryo tissue generation, tissue regeneration after wounding, and is implicated in the development of cancer [14]. OSE cells normally undergo EMT to heal the wound that forms following ovulation. Uncommitted OSE cells normally express keratin, which is associated with an epithelial cell type [12]. However, these cells also constitutively express vimentin, N-cadherin, and smooth muscle alpha-actin, all of which are associated with the mesenchymal phenotype [12]. OSE cells also produce several proteolytic enzymes (which help to degrade the epithelial cell wall during ovulation), as well as secrete collagen type III, characteristics that are also common to mesenchymal cells. OSE cells express low levels of the mucin MUC16 (CA125). Mullerian-duct derived tissues express high levels of MUC16 (CA125), as do ovarian tumors [15]. As we will discuss later, MUC16 (CA125) over expression in ovarian tumors is an important marker for progression and regression of EOC.

OSE cells undergo EMT transition after ovulation to remodel the extracellular matrix and repair the post-ovulatory wound that is generated during expulsion of the oocyte. Epithelial cells are characteristically polar and are bound together with molecules (such as E-cadherin) that facilitate cell-cell junctions. Conversely, the mesenchymal phenotype is that of motility and movement, as well as reduced polarity of a cell [16]. The transition of OSE to a mesenchymal phenotype aids in the ovulatory process because these converted cells have increased motility, altered proliferative responses, and the ability to remodel the extracellular matrix (ECM) [17]. TGF-β, EGF, and collagen are all present at the site of ovulatory rupture and can induce OSE EMT. OSE cells also undergo EMT in collagen matrices. It is a normal function of OSE to undergo EMT, therefore, cancer may represent unregulated EMT [14].

The expression of markers that are associated with those of Mullerian-duct derived tissue are found in inclusion cysts, which are the site of many neoplasms. OSE lining inclusion cysts express higher levels of EOC markers MUC16 (CA125) and CA19-9 and is two to three times more metaplastic in women with ovarian tumors compared to OSE in normal ovaries [18]. The hypothesis that EOC may derive from inclusion cysts is based upon the incessant ovulation theory, first proposed by Fathalla in 1971 [19]. This theory is based upon epidemiological data that reveals that women on birth control or who have been pregnant and/or breastfeeding have decreased risk of ovarian cancer. Fathalla suggested that wounds in the epithelium surrounding the ovary caused by ovulation month after month can cause increased inflammation and cell proliferation; thereby increasing the chance for cells to form neoplasms. Higher ovulatory activity is associated with an increased accumulation of inclusion cysts and invaginations of the OSE, which provide a hospitable environment for tumor cell growth [20]. This concept is supported by in vitro evidence in which ovarian surface epithelial cells from both rats and mice have been continuously cultured, mimicking the constant damage and repair that OSE undergo. In both species these *in vitro *cells spontaneously transformed into cancerous cells [21-23]. Another observation that supports the incessant ovulation hypothesis is that studies have repeatedly shown that oral contraceptive use (which prevents ovulation) reduces ovarian cancer risk [24].

An alternative hypothesis related to that of incessant ovulation is known as the gonadotropin hypothesis [25-27]. High levels of gonadotropins initiate each ovulation and persist immediately after menopause. These hormones stimulate the ovulation-like process involving the expression of cytokines and proteolytic enzymes within the surface epithelium. I nflammatory factors may lead to a loss of the basement membrane and the formation of inclusion cysts which can contribute to cell transformation into cancer [20]. One animal model (ewes) showed that oxidants released during ovulation caused DNA fragmentation and apoptosis in cells that were closest to the rupture, while milder DNA damage and the accumulation of p53 was shown in decreasing levels farther away from the rupture site [28].

Others hypothesize that ovarian tumors do not arise from OSE at all, but derive directly from the Mullerian-duct tissues and migrate to the ovarian surface. Dubeau first proposed this hypothesis in 1999 [29]. According to Dubeau, the theory which suggests that OSE cells must first differentiate into Mullerian-duct type cells via metaplasia before becoming neoplastic contradicts our current understanding of cancer, which is that the cancerous cells are less differentiated than the cells they originate from [30]. He suggests that a more likely scenario is that EOC derives from Mullerian-duct derived tissues, and has several compelling observations to support this hypothesis. Ovarian tumor cells share many similar characteristics to the cells of the fallopian tubes, uterus, and endocervix, and do not share histological or protein expression profile with the OSE. Dubeau argues that the fimbrae of the fallopian tubes, which literally rub up against the surface of the ovary during ovulation and sometimes adhere to the surface of the ovary due to inflammation, are a prime site for the development of metaplasia. The cells from the fimbrae of the fallopian tubes have been shown to have developed pre-neoplastic changes in women who have undergone surgery for prophylactic removal of their fallopian tubes because of a mutation in BRCA1 [31-33].

In addition to histological changes found in the fimbrae of the fallopian tubes, mutations in the tumor suppressor gene p53 in the distal fimbrae of women with the BRCA^+ ^mutation have also been observed [34]. Christopher Crum's group found strong p53 staining in benign tissues from BRCA^+ ^women who underwent prophylactic salpingo-oophorectomies. This staining correlated with mutations in the p53 gene in these same cells. Because the p53 mutations were found predominantly in the distal fimbrae of the fallopian tubes (the cells that are in contact with the OSE), the location of this staining may reveal one mechanism by which ovarian tumors arise in BRCA^+ ^women [34]. In 2008, Crum's group correlated the p53 mutation in the fallopian tube fimbrae with lower parity and increased age at first childbirth, which links this marker to incessant ovulation [35]. A comparison of p53 mutations in ovarian inclusion cysts with p53 mutations in the fimbrae of fallopian tubes, again from women who were BRCA^+ ^was conducted. The results revealed that p53 mutations were not present in any inclusion cysts that were examined, but were present in 38% of fimbrae of fallopian tubes from these women [36]. Another piece of evidence to support the argument that EOC arises from the fallopian tube is that several studies have shown that tumor cells clinically identical to ovarian cancer cells are found in the peritoneal environment in women years after their ovaries have been removed for reasons other than cancer [37-39].

Dubeau states that ovarian cancer is over-diagnosed, and many of these cancers actually arise from the fallopian tube or peritoneal cavity wall. The origin of ovarian tumors is of important consideration, not only for nomenclature reasons, but for women who have the BRCA1 or BRCA2 mutation and are undergoing prophylactic surgery and who want to preserve their fertility. If the origin of ovarian cancer is indeed not the ovary, then the ovaries need not be removed, and cryopreservation of oocytes for future use is not an issue [30].

## Ovarian cancer detection

Attempts to find an accurate screening test for EOC have, to date, been unsuccessful. CA125 (MUC16), originally thought to be an indicator of ovarian cancer, is now known to be quite non-specific as well as to lack the sensitivity to detect stage I disease. Bast and coworkers showed in the 1980s that CA125 was expressed in the serum of the majority of patients with EOC, as well as patients with cancer of the endometrium, fallopian tube, and endocervix [40-44]. CA125 serum levels are elevated in 80% of advanced stage EOC patients; however, this marker can be elevated in a variety of benign conditions and other non-gynecologic malignancies. High concentrations are found in pancreatic, breast, bladder, liver, and lung cancers, as well as benign diseases such as diverticulitis, uterine fibroids, endometriosis, benign ovarian cyst, tubo-ovarian abscess, hyperstimulation syndrome, and ectopic pregnancies [42,45-48]. Elevated levels are also found in physiological conditions including both normal pregnancy and menstruation [49]. Furthermore, CA125 levels are elevated in less than half of the cases in early-stage ovarian cancers, underscoring the lack of sensitivity to diagnose curable disease. Therefore, CA125 is not used as a screening test, but mainly as a measure of disease progression, regression, and predictor of recurrence during treatment for EOC. CA125 levels measured over a period of time along with transvaginal sonography has been shown to increase sensitivity [50], however, the cost of transvaginal screening limits its use in the general population. CA125 itself is a repeating peptide epitope on the large molecular weight mucin, MUC16 [51-54]. This mucin is expressed at low levels by normal ovarian surface epithelium and is overexpressed by EOC tumor cells [43,49]. Tumor cells secrete MUC16 into the peritoneal fluid (PF) and from the abdominal cavity this mucin leaks into the blood stream and can then be detected via the CA125 serum assay.

Proteomic approaches are being utilized to identify molecular markers for ovarian cancer and mathematical models are being developed to identify specific patterns that are indicative of disease [55]. Other promising markers for ovarian cancer include human epididymis protein-4 (HE4), decoy receptor-3 (DcR3), osteopontin, mesothelin, spondin-2, SMRP, CA72-4, ERBB2, inhibin, activin, EGFR, and lysophosphatidic acid, [50,56-66]. Of these the most promising is HE4 which is expressed on ovarian tumor cells from some patients that do not express CA125. Indeed, studies have shown that the combined monitoring of serum levels of CA125 and HE4 is likely to significantly improve the sensitivity for detection of ovarian cancer in women with pelvic mass [67]. An important study published recently has concluded that a steady increase in the serum concentrations of CA125, HE4, and mesothelin can be detected in patients up to 1-3 years before a clinical diagnosis of ovarian cancer is made in patients [68].

## Ovarian cancer staging and treatment

Ovarian cancer is a surgically staged disease, meaning that it is impossible to tell what the stage of the cancer is without examining the extent of the metastasis surgically. Metastasis of ovarian cancer spreads by direct extension to neighboring organs from the ovaries or by the sloughing of tumor cells into the peritoneal cavity. These individual or groups of exfoliated cells float in the fluid of the peritoneal cavity and can subsequently bind to the wall of the peritoneal cavity and form additional lesions. The tumor cells also commonly disseminate by lymphatic spread [69]. Proper surgical staging requires a complete inspection of the peritoneal cavity and its contents, as well as evaluation of the retroperitoneal spaces and lymph nodes. At the same time that the EOC patient is being evaluated for the stage of the disease, the surgeon also attempts to remove all visible tumors from within the peritoneal cavity. Additionally, the surgeon washes the peritoneal cavity several times with saline in order to remove as many tumor cells as possible. This procedure is termed cytoreductive surgery or tumor debulking [70].

The stages (I-IV) of ovarian cancer are determined by the extent of metastasis. Stage I EOC is confined to the ovaries whereas stage II affects other pelvic structures. In stage III, the disease has spread beyond the pelvis into the upper abdominal cavity or into the draining nodal beds irrespective of peritoneal based disease. Stage IV is defined as disease outside of the peritoneal cavity and most commonly includes parenchymal liver lesions or malignant pleural effusions. Patients with stage I disease most commonly undergo bilateral oophorectomy, hysterectomy, and surgical staging including peritoneal biopsies, omentectomy, and pelvic and aortic lymph node dissection. In select cases of younger patients who wish to preserve fertility, only the affected ovary may be removed and a hysterectomy would not be performed [70]. Chemotherapy treatment in early stage disease is dependent upon the grade of the tumor. It is recommended that patients with advanced stage (II, III or IV) EOC undergo cytoreductive surgery to remove all visible tumor whenever feasible, followed by platinum and taxane based chemotherapy [70]. Despite a high rate of initial remission, these patients have a high rate of recurrence (at least 50%) and overall poor survival. Cancer diagnosed in early stages has a much higher 5-year survival rate (Stage I: >90%, Stage II: 70-80%) compared to cancer diagnosed in later stages (Stage III: 20-30%, Stage IV: <5%) [70]. A major advance in the treatment of ovarian cancer has come from intraperitoneal administration of platinum and taxane agents instead of the more conventional intravenous delivery of these drugs [71-73]. Of the 654 randomized patients included in one trial, the median survival for patients receiving intraperitoneal cisplatin was 49 months compared to 41 months for the cohort receiving intravenous cisplatin [73]. Increased cytotoxicity remains a major hurdle curtailing the efficacy of intraperitoneal chemotherapy [74].

Treatment is made difficult for EOC patients because metastasis is acute and tumor cells exert immunosuppressive effects. The anatomical location of the ovaries within the peritoneal cavity facilitates metastasis because tumor cells can spread by sloughing off of the main tumor and binding to many organs in the vicinity, including the peritoneal cavity surfaces and the highly vascular omentum [75]. This complicates treatment in that it is technically impossible to remove all cancerous cells during cytoreductive surgery. The accumulation of peritoneal fluid in ovarian cancer patients also contributes to metastasis by aiding the flow of tumor cells within the peritoneal cavity. Peritoneal fluid contains secretions from the tumor cells that have now been shown to contain many factors which aid in the inhibition of the immune system in these patients [76-83]. Furthermore, ovarian tumors also acquire resistance to chemotherapy. Spheroids, or clumps of tumor cells (extremely common in the peritoneal fluid of EOC patients), have been shown to be more resistant to chemotherapy [84]. De novo and acquired chemoresistance combined with expression of immunosuppressive factors makes it difficult to effectively treat ovarian cancer [85,86].

## Tumorigenesis and Metastasis

Tumorigenesis requires several genetic alterations, either somatic or inherited, that confer a selective growth advantage to the neoplastic cell population. During tumor development, initial random genetic alterations result in a tumor cell population with a proliferative advantage. These tumor cells become the progenitors of a clonal population that eventually dominates the tumor mass. Tumor progression is analogous to Darwinian selection, with repeated mutations and subsequent dominance of the daughter cell population via expression of traits that confer a survival advantage [86].

A defining characteristic of a malignant epithelial tumor is invasion beyond the basement membrane into the surrounding stromal tissues. For example, in breast disease benign tumors such as fibrocystic lesions, sclerosis adenoma, and fibroadenoma are all characterized by disorganization of the normal epithelial architecture. However, no matter how extensive this disorganization may become, these benign lesions are always characterized by a continuous basement membrane that separates the neoplastic epithelium from the stroma [87]. Malignant tumors are characterized by their ability to invade through the basement membrane after which it is impossible to determine how many cells have escaped from the primary tumor and have established at metastatic sites [88]. Similar to malignant invasion some non-cancerous cells can physiologically invade basement membranes. Common examples of this include migration of immune cells during an inflammatory response, endothelial cells during an angiogenic response, and trophoblasts into the endometrial stroma and blood vessels to establish contact with the maternal circulation during placentation. The mechanisms used by these cells are thought to be very similar to those used by invading tumor cells [88,89]. The difference between these normal functions and the invasion associated with tumor cells is the lack of regulation seen in cancer. The mechanisms for the regulation of invasiveness are yet undetermined. Development of novel therapeutic agents towards these factors could help treat inflammatory, and angiogenesis disorders, as well as cancer formation [88].

Once a tumor is established metastasis may occur. While primary tumors are usually successfully eliminated by surgical or chemotherapeutic means, metastases are more difficult to detect and treat [89]. Metastases can cause death via paraneoplastic syndromes, interference with the normal functioning of an organ because of a growing lesion, or from complications related to treatment [89].

EOC was originally thought to be of the linear-clonal model of metastasis, which states that a late stage clone of the tumor acquires an additional genetic change that enables metastatic progression [90]. However, metastasis may not be the final stage of clonal evolution during tumor progression. Some cells seem to have derived from early stage clones in the primary tumor while others derive from later stage clones. This group supports a model in which primary ovarian cancers have a common clonal origin but become polyclonal with different clones at both early and late stages of genetic divergence acquiring the ability to progress to metastasis [90].

The complexity of metastasis increases when one considers that each cancer type typically metastasizes to different areas in the body. This is termed the "seed vs. soil" hypothesis which was first observed by Stephen Paget in 1889 [91]. Referring to the tumor cell as the seed and a potential metastatic site as the "soil," he stated, "When a plant goes to seed, the seeds are scattered in all different directions; but they can only live and grow if they land on congenial soil." He hypothesized that this theory could be used to predict metastatic locations for different cancers. Different selective pressures exist in different organs and the tumor cells must adapt to these environments. Some of these pressures include hypoxia, presence of reactive oxygen species, or lack of nutrients. Tumor cells must then alter their phenotype in order to exist in environments with different selective pressures [92].

In ovarian cancer, the "seed vs. soil" observation holds true as the most common sites of metastasis are within the peritoneal cavity. Mesothelial cells that express mesothelin line the walls of the peritoneal cavity as well as the organs within it. We and others have shown that MUC16, present on the surface of cancer cells, binds readily to mesothelin [93,94]. Recently, the binding site for MUC16 on mesothelin was characterized [95]. This interaction is just one of the many that make the "soil" of mesothelial cells within the peritoneal cavity an appropriate environment for ovarian cancer tumor cells.

In order to efficiently metastasize, tumor cells must first detach from the primary tumor by downregulating adhesive molecules, then later upregulate adhesive molecules to attach again to the target site epithelium. The initial step of detachment requires disruption of cell-cell adhesions, and this is facilitated by a loss of E-cadherin. E-cadherin is tethered to the actin cytoskeleton, which plays a primary role in supporting cell-to-cell adhesions. The disruption of the expression of E-cadherin can then lead to cells which can disseminate from the primary tumor. Loss of E-cadherin function is necessary but not sufficient for an epithelial to mesenchymal cell type transition [95]. Loss of E- cadherin has been seen in many types of cancers, such as breast, prostate, esophagus, stomach, colon, skin, kidney, lung, liver, and ovary [96,97].

After detachment from the primary tumor site, the next step of metastasis is to effectively invade into neighboring tissues. Movement of the tumor cells through solid tissues requires the acquisition of phenotypes that allow cells to degrade the ECM and subsequently acquire forward propelling movements to invade into these tissues [92].

Next, the tumor cells migrate into the circulation, lymphatic system, or peritoneal space. In EOC, metastasis is facilitated by the clockwise flow of peritoneal fluid. The final steps of metastasis include arrest in the small blood vessels of a distant organ, extravasation into the surrounding tissue and proliferation at the secondary site [92].

## Immune Evasion

Patients with EOC often experience several periods of remission and relapse of increasingly shortening periods until their tumors become resistant to chemotherapeutic treatment [98]. Additionally, as the stages of cancer progress, patients exhibit progressively deficient immune responses, which indicate that the tumor has developed mechanisms to subvert the immune response and suppress immune surveillance [99]. The importance of the role of the immune system in the control and elimination of EOC is evidenced by a study that correlated the 5-year overall survival in EOC with the presence or absence of tumor-infiltrating lymphocytes (TIL) (38% vs. 4.5%, respectively) [100]. There are several studies which show that molecules from the tumor directly inhibit immune cells. We have now also demonstrated that MUC16 protects the ovarian tumor cells by sterically blocking the NK cells from forming immune synapses with the cancer cells [101]. High levels of shed MUC16 (sMUC16) are present in the PF of EOC patients and this mucin binds to NK cells within the PF [76]. MUC16 binds specifically to the inhibitory receptor, Siglec-9 on the surface of the NK cells (Belisle et al., paper submitted). Normally, NK cells in the peripheral blood of healthy subjects have the phenotype 90% CD16^+ ^and 10% CD16^-^. The CD16^+ ^phenotype is associated with activation and cytotoxicity, while the CD16^- ^cells release cytokines and are not cytotoxic [102]. In the PF of EOC patients, however, this ratio shifts to 60% CD16^+ ^and 40% CD16^-^. Therefore, there are less cytotoxic cells in the PF compared to the peripheral blood [76].

Other immune cell subsets can also be affected by factors within peritoneal fluid. A study published in 2001 described a factor within PF that induced the loss of the T cell receptor (TcR)-associated signal transducing zeta-chain (CD3ζ) [81]. They isolated this factor using column chromatography, gradient centrifugation, and mass spectrometry and found that it was a 14 kD factor that operated at the mRNA level [81]. Webb and colleagues have shown that CD1d antigen presentation to NKT cells is inhibited by factors within the PF. This effect was dose dependent and CD1d specific [103]. Another study determined that supernatants from ovarian cancer cell lines inhibited CD8^+^T cell proliferation and function, as well as the cells' ability to produce IFN-γ. IL-2R subunits γ and β (but not α) were significantly suppressed as measured by flow cytometry [104]. Our group has also described the presence of Decoy Receptor 3 (DcR3) in the peritoneal cavity of women with advanced EOC and that this molecule functions as a potent inhibitor of Fas-ligand mediated apoptosis a common regulatory mechanism of the normal immune system [80].

Tumor cells also produce ligands that can bind to activating receptors on immune cells and thus downregulate the expression of these receptors. The ligands for activating receptor NKG2D are MHC class I-chain-related proteins A and B (MICA/MICB) and the UL16-binding proteins (ULBP-3) [105]. NKG2D ligands are not expressed on normal, healthy cells and therefore the expression of NKG2D ligands is correlated with malignant transformation. NKG2D receptor is expressed by all NK cells, CD8^+ ^T cells, most NKT cells, and a subset of CD4^+ ^T cells [105]. When NKG2D binds to its ligands, it induces the cytotoxic activation and proliferation of the immune cell. However, MICA and MICB can be cleaved from tumor cells by tumor-associated mellatoproteinases, which leads to soluble MICA and MICB that can downregulate the expression of NKG2D [106]. Wang and colleagues showed, using flow cytometry, that serum from prostate and ovarian cancer patients contained high levels of soluble MICs and correlated increased soluble MIC expression with decreased expression of NKG2D on T cells and a subset of NKT cells in these patients [107]. Another study used immunohistochemistry to determine that tumor from 82 ovarian cancer patients showed expression of MICA, MICB, and ULBP-2, while none of these molecules was expressed by normal ovarian epithelium [108]. Strong expression of ULBP-2 correlated with decreased infiltration of T cells and poor prognosis [108].

## Immunotherapy In EOC

Most pre-clinical models of cancer immunotherapy indicate that such treatments work best in the setting of minimal volume, sub-clinical disease. Thus it is thought that patients with minimal residual disease who clinically appear to be in remission are ideal candidates for immunotherapeutic strategies. Immunotherapies may not be robust enough to eliminate the entire tumor when used alone, however; their use after surgery and chemotherapy may be useful to eliminate remaining sub-clinical tumor cells to prevent recurrence. The high rate of clinical response to therapy and the subsequent high rate of recurrence in EOC after primary treatment is evidence of a large number of women with sub-clinical disease at the completion of therapy. These patients may offer an excellent setting for immunotherapy.

There are several immunotherapies that have been targeted to MUC16 as well as mesothelin. One such immunotherapy, oregovomab, is an immunoglobulin (Ig) I gG1k subclass murine monoclonal antibody that binds with high affinity to circulating CA125. This antibody complexes with CA125 and is taken up and processed by APCs (antigen presenting cells) [109,110]. Both a humoral and cellular response are produced, as demonstrated by the production of CA125 specific antibodies, T-helper cells, and CTLs in patients who received treatment [109,111,112]. Survival was increased in patients that mounted T-cell responses against CA125, however, the most recent results from a phase III trial published in January of 2009 stated that monoimmunotherapy treatment with oregovomab resulted in no significant difference in outcome compared to placebo [111].

Antibodies, designated 3A5 and 11D10, against the tandem repeat sequence of MUC16 have been conjugated to the cytotoxic auristatin analogs monomethylauristatin F and monomethylauristatin E [113,114]. These drug-conjugated antibodies have been utilized as agents for chemotoxic immunotherapy resulting in an improved therapeutic index against MUC16-expressing OVCAR-3 tumors that were xenogenically grown in mice [113].

Abagovomab (ACA125) is an anti-idiotypic antibody against the MUC16 antibody OC125 and mimics the antigenic epitope of MUC16. It serves as a surrogate when given to patients. In phase I and II trials, patients that received abagovomab antibody developed anti-anti-idiotypic antibodies (Ab3) and this correlated with increased survival [115,116]. Reinartz and colleagues developed a fusion protein of ACA125 with interleukin 6 in order to stimulate ACA125 specific B cells [117]. This resulted in increased levels of Ab3 in patients who received treatment.

Mesothelin is normally expressed by mesothelial cells that line the pleura, peritoneum, and pericardium. It is highly expressed by tumor cells associated with pancreatic, ovarian, and lung adenocarcinomas as well as malignant mesothelioma [118,119]. Its normal function is unknown and knockout mice show no abormalities [120]. However, we and others [93,94] have shown that it binds to MUC16, which facilitates the metastasis of ovarian cancer cells to the peritoneal cavity. Agents that would inhibit this interaction would be beneficial to prevent metastasis in EOC patients. A majority of patients with serous epithelial ovarian cancer show increased levels of serum mesothelin, making it a suitable target for immunotherapies, considering its relatively low expression in normal tissues [121]. SS1P is a recombinant immunotoxin consisting of an anti-mesothelin Fv linked to a *Pseudomonas *exotoxin that mediates cell killing. Phase I trials have been completed with SS1P and have shown anti-tumor activity in heavily treated patients [122]. Pre-clinical studies in animal models have shown that treatment with SS1P has an increased effect when combined with chemotherapy [123].

MORAb-009 is a high affinity chimeric monoclonal IgG1/κ with high affinity and specificity for mesothelin [124]. This antibody both induces ADCC (antibody-dependent cellular cytotoxicity) against tumor cells that express mesothelin as well as blocks the MUC16/mesothelin interaction [124,125]. Phase I trials with MORAb-009 are underway with 11 patients, 6 with mesothelioma, 3 with pancreatic cancer, and 2 with ovarian cancer. CRS-207 is another mesothelin cancer vaccine that utilizes *Listeria monocytogenes *as the vector. Pre-clinical studies have shown this vaccine to elicit CD4^+^/CD8^+ ^T cell mesothelin specific responses in mice and cynomolgus monkeys. A Phase I trial for CRS-207 is underway [123].

There are several other molecular candidates that are being investigated for immunotherapy against ovarian cancer. Incubation of immune cells with ovarian cancer cells lead to generation of antigen specific T cells against THP-1 and other peptide epitopes of ovarian cancer [126]. Other potential antigens for immunotherapy include p53, Her-2 and TPD52. Vaccination with Her-2 peptides along with measles virus fusion protein, a promiscuous T cell epitope causes increased anti-tumor immune responses [127]. Similarly, 66% of mice developing responses against TPD52 expressing prostate tumors were free of the cancer 85 days after tumor inoculation and were also able to resist a subsequent tumor challenge [128]. The high expression of TPD52 by ovarian tumors provides hope that this strategy may also provide benefit to ovarian cancer patients.

Autoantibodies against p53 are present in ovarian cancer patients and their presence is associated with improved survival [129]. In a phase II clinical study, patients vaccinated against specific p53 peptides showed proliferation of p53 specific T cells [130]. These proliferating T cells were immune competent and produced high levels of IFN-γ. A subset of the patients (2/20; 10%) developing p53-specific T cells showed evidence of stable disease as compared to the remaining cohort with clinical and biochemical evidence of progressive disease. These data indicate that more research is required to produce effective immunotherapeutic approaches for the treatment of ovarian tumors.

## Conclusion

Cytoreductive surgery followed by intense chemotherapy with platinum and taxol has become a standard approach for the treatment of EOC. Therapy is especially effective if the cancer is detected at early stage of progression. Future advances in the management and cure of EOC will depend on development of novel treatment modalities and diagnostic tests that can accurately detect early stage low volume tumors. While chemotherapeutic approaches have been important in the management of EOC, there is a growing sense in the field that additional supportive therapeutic approaches will be required for effective elimination of the cancer. The polyclonal nature of EOC ensures that therapeutic approaches may not eliminate the entire spectrum of cancer cells present in a patient. Combinatorial approaches that can result in direct cytotoxicity, prevent tumor angiogenesis, inhibit cancer metastasis, and also simultaneously increase the immunologic detection of tumors may be required to eliminate the polyclonal tumors. Such a holistic approach will require delineation of the molecular mechanisms that allow tumors to metastasize, promote angiogenesis, and to circumvent any effective immunological responses.

The combined treatment strategies will benefit from the development of diagnostic and screening tests. To date the "gold standard" for assessing the regression and recurrence of EOC is the serum CA125 (MUC16) assay. However, this assay is limited in its scope. Development of novel proteomics based approaches for the development of diagnostic tests hold great promise. However, even after intense research, successful development of a proteomics-based diagnostic test has remained elusive.

Overall, significant hurdles still remain in the effective diagnosis and treatment of EOC. The significant advances made in the molecular understanding of EOC, development of murine models and novel proteomics-based technologies, and the use of immune-based treatment approaches are likely to provide novel opportunities for the effective management of EOC.

## Competing interests

The authors declare that they have no competing interests.

## Authors' contributions

JAAG, JPC, and MSP did the majority of the writing of this manuscript. NC and AK contributed by writing specific sections of this manuscript. All authors have read and approved this manuscript.
